# Comprehensive Analysis of Factors Associated with New Episode of Postoperative Atrial Fibrillation after Coronary Artery Bypass Graft Surgery

**DOI:** 10.3390/life13102035

**Published:** 2023-10-10

**Authors:** Olesya Rubanenko, Anatoly Rubanenko, Igor Davydkin

**Affiliations:** 1 Hospital Therapy Department, Samara State Medical University, 89, Chapaevskaya St., 443099 Samara, Russia; i.l.davydkin@samsmu.ru; 2 Propaedeutic Therapy Department, Samara State Medical University, 89, Chapaevskaya St., 443099 Samara, Russia; a.o.rubanenko@samsmu.ru

**Keywords:** atrial fibrillation, coronary artery bypass graft, inflammation, oxidative stress, fibrosis, myocardial dysfunction, myocardial ischemia, omega-3 index

## Abstract

The aim of the study was to perform a comprehensive fundamental analysis of the factors of inflammation, oxidative stress, fibrosis, myocardial dysfunction, ischemia and omega-3 index associated with postoperative atrial fibrillation (POAF) after coronary artery bypass graft (CABG) surgery in patients with coronary artery disease. The study involved 158 patients who were admitted to the hospital to undergo CABG surgery. Patients were divided into two groups: group 1 comprised 111 patients without POAF (82% males, median age—62.0 (56.0; 66.0) years), and group 2 comprised 47 patients with POAF (84.4% males, median age—65.0 (61.0; 70.0) years). POAF occurred 5.2 (2.0; 7.0) days after CABG. In all the patients, we evaluated laboratory tests before and 3–4 days after CABG. All the patients also underwent echocardiography. According to results of multifactorial regression analysis, the odds ratio of POAF development for left atrial diameter >41 mm was 4.3 (95% confidence interval (CI) 2.0–9.7, *p* < 0.001), interleukin (IL)-6 postoperative levels >22.07 pg/mL—3.0 (95% CI 1.4–8.2, *p* = 0.006), IL-8 postoperative levels >9.67 pg/mL—2.3 (95% CI 1.2–7.3, *p* = 0.006), superoxide dismutase postoperative levels in plasma >1100.5 U/g—3.2 (95% CI 1.4–9.2, *p* = 0.03), glutathione postoperative levels ≤0.194 micromole/g of hemoglobin—1.9 (95% CI 1.2–6.3, *p* < 0.001), glutathione peroxidase postoperative levels ≤17.36 millimole/g of hemoglobin—2.2 (95% CI 1.1–8.2, *p* < 0.001), glutathione reductase postoperative levels ≤2.99 millimole/g of hemoglobin—2.3 (95% CI, 1.1–5.7, *p* < 0.001), malondialdehyde postoperative levels >1.25 micromole/g of hemoglobin—2.0 (95% CI, 1.2–7.9, *p* < 0.001), NO postoperative levels in plasma >36.4 micromole/L—1.5 (95% CI, 1.1–5.9, *p* < 0.001) and omega-3 index ≤1.59%—2.6 (95% CI 1.5–9.1, *p* < 0.001). Our study showed that increased left atrial diameter, high postoperative levels of inflammatory factors, oxidative stress, fibrosis indicators and omega-3 index were associated with POAF in patients who underwent CABG.

## 1. Introduction

Cardiac surgery, particularly coronary bypass graft surgery (CABG), in patients with coronary artery disease (CAD) improves the quality of life, reduces the severity of myocardial ischemia and, in some patients, prolongs life [1,2]. Intraoperative and postoperative periods are accompanied by hemodynamic overload for the myocardium, as they lead to an increase in inflammation and oxidative stress, hemostatic disorders, electrolyte imbalance and fluid overload [3]. Therefore, searching for methods to reduce the risk of early and late postoperative complications in this group of patients remains necessary.

Atrial fibrillation (AF) in patients with CAD remains a common complication of coronary artery bypass graft (CABG) surgery with prevalence up to 40% [4]. Postoperative AF (POAF) is associated with an increased risk of stroke and heart failure (HF), early and late mortality and prolonged hospital length of stay [4]. The occurrence of AF in the early postoperative period also increases the risk of death and postoperative consciousness disorders [4].

Moreover, postoperative AF is significantly associated with postoperative acute kidney injury [5].

Previous comorbidity, surgical intervention accompanied by myocardial damage due to ischemia and reperfusion and changes in atrial pressure as a result of ventricular dysfunction due to stunning and autonomic dysfunction are provoking factors of arrhythmia [6]. However, the molecular and cellular mechanisms of the occurrence and maintenance of POAF are not well known.

The aim of our observational prospective study was to perform a comprehensive fundamental analysis of the factors of inflammation (interleukin (IL)-6, IL-8, IL-10, C-reactive protein (CRP)), oxidative stress (superoxide dismutase (SOD), reduced glutathione (RG), glutathione peroxidase (GPO), glutathione reductase (GR), malondialdehyde (MDA)), fibrosis (metalloproteinase (MMP)-9), myocardial dysfunction (N-terminal pro-B-type natriuretic peptide (NT-proBNP)), ischemia (troponin I) and omega-3 index associated with new-onset AF after CABG surgery in patients with CAD.

## 2. Materials and Methods

A total of 158 patients with CAD who underwent CABG were enrolled in an observational prospective study in Samara Regional Clinical Cardiology Dispensary, named after V.P. Polyakov, from January to June 2015.

Inclusion criteria: stable CAD and signed informed consent. Exclusion criteria: mechanical heart valve prosthesis or valvular heart disease that requires valve replacement surgery, acute liver and renal diseases, cancer, acute stroke or transient ischemic attack, coagulopathy, history of AF, thyroid gland diseases and age over 80 years.

All the patients underwent standard instrumental and laboratory tests. Transthoracic echocardiography was performed using ultrasound scanners Logiq—5 and 7 (General Electric company, Boston, USA) in M, B, D-modes. CABG was performed on-pump or off-pump. After median sternotomy, routine unicaval two-stage venous cannulation and aortic cannulation was performed for cardiopulmonary bypass. After ante- and retrograde cardioplegia protocol, cross-clamp was placed. The primary study end point was new-onset POAF.

Patients were divided into 2 groups: group 1—patients without POAF (111 patients, 82.0% men, median age 62.0 (56.0; 66.0) years); group 2—patients with new-onset AF after CABG (47 patients, 84.4% men, median age of 65.0 (61.0; 70.0) years). POAF was registered during monitoring in the intensive care unit and using ECG.

Interleukin (IL)-6, IL-8, IL-10, C-reactive protein (CRP), superoxide dismutase (SOD), reduced glutathione (RG), glutathione peroxidase (GPO), glutathione reductase (GR), malondialdehyde (MDA), N-terminal pro-B-type natriuretic peptide (NT-proBNP), troponin I, omega-3 polyunsaturated fatty acids and metalloproteinase (MMP)-9 levels were estimated on admission and after 3.0 (3.0; 4.0) days after surgery. The levels of laboratory tests were estimated using the immunoassay technique on Thermo Scientific Multiscan FC microplate photometer (Thermo Fisher Scientific, Massachusetts, USA) with the following test systems: IL-6, IL-8, IL-10, NT-proBNP and CRP enzyme-linked immunosorbent assay (ELISA kit)—BEST («Vector-BEST», Novosibirsk, Russia), ELISA—SOD («Cytokine», Saint-Petersburg, Russia). Troponin levels were measured with the immunoassay system UNICEL^®^ DXI 600 ACCESS (Beckman Coulter, Brea, CA, USA).

Indicators of SOD in erythrocytes, MDA, the level of RG and GR, and GPO were calculated using a spectrophotometric method on a spectrophotometer LOMO SF-56 (OKB Spectrum, St. Petersburg, Russia). The omega-3 polyunsaturated fatty acids were determined via chromatographic analysis. When identifying and quantifying MMP, the method of zymography in polyacrylamide gel with copolymerized gelatin was used.

Descriptive statistics was carried out using Statistica 7.0 software (StatSoft Inc., St Tulsa, OK, USA). Quantitative variables are shown as median, 25 and 75 percentiles, and qualitative variables are shown as absolute numbers and percentages of the total patients with the available data for each group. Univariate and multivariate logistic regression analysis were used to identify factors associated with POAF. Statistically significant *p*-values were considered to be less than 0.05.

By prospectively setting the “α err prob” value to 0.05, “effect size d” value to 0.3 and “power (1-β err prob)” value to 0.8, and conducting a power analysis with sample-size.net, we found that this study should include at least 114 patients. In our study, the total of 158 patients was enough to meet the requirement of high efficiency (0.3).

## 3. Results

**Figure 1:**
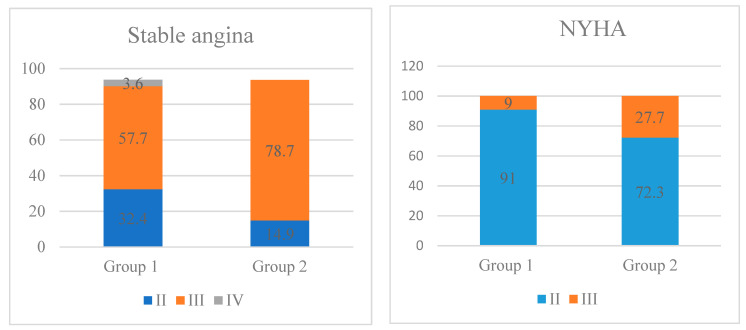
The incidents of stable angina and NYHA, % of patients.

During the observational period, AF occurred in 29.7% of cases, on average 5.2 (2.0; 7.0) days after surgery. Clinical and instrumental indicators of patients are shown in Table 1 and Figure 1.

Patients in group 2 were older (65.0 (61.0; 70.0) vs. 62.0 (56.0; 66.0) years old, *p* = 0.008), had stable angina III more often (37 (78.7%) vs. 64 (57.7%), *p* = 0.012), NYHA III (13 (27.7%) vs. 10 (9.0%), *p* = 0.003) and had a longer history of CAD (60.0 (13.5; 138.0) vs. 15.5 (8.0; 72.0) months, *p* = 0.01).

In our study, we estimated echocardiographic and surgical indicators (Table 2).

Patients in group 2 had larger left atrial (LA) diameter (44.0 (40.5; 46.0) mm vs. 38.0 (36.0; 40.0) mm, *p* < 0.001) and LA volume (55.9 (48.1; 63.1) mL vs. 44.1 (41.9; 45.4) mL, *p* < 0.001) compared with group 1. There were no significant differences between patients of both groups according to other indicators.

In group 2, there was an increase in the time of extracorporeal circulation and aortic cross-clamping time, but the data did not reach statistical significance, which may be because of the small number of patients.

In 30 (63.8%) patients, one episode of AF was recorded, in 6 (12.8%)—two episodes, in 6 (12.8%)—three episodes, in 4 (8.5%)—four episodes, and in 1 patient (2.1%)—five episodes. In 36 (76.6%) cases, POAF was stopped by the introduction of a class III antiarrhythmic drug (amiodarone), in 6 (12.8%) cases—by electrical cardioversion, and in 5 (10.6%) cases—stopped without treatment. To assess the symptoms associated with arrhythmia, the class of modified symptom scale for AF EHRA (European Heart Rhythm Association) was taken into account: class 1 was observed in 3 (6.4%) patients, class 2a—in 6 (12.8%), class 2b—in 26 (55.3%), class 3—in 10 (21.3%), and class 4—in 2 (4.2%) patients.

It should be noted that one (0.9%) patient in group 1 and 3 (6.4%) of group 2 developed stroke, and two patients from them were transferred to the neurological department for further treatment.

In one patient in group 2, the postoperative period was complicated by sick sinus syndrome, which required the implantation of a pacemaker. The patient underwent coronary catheterization, and grafts were normal.

In patients in group 2, in the preoperative period, IL-6 (5.5 (3.5; 46.0) pg/mL vs. 3.6 (2.6; 15.6) pg/mL, *p* = 0.03), SOD (2751.8 (1563.0; 3949.8) units/g vs. 1775.8 (924.8; 4718.8) units/g, *p* = 0.04), RG (0.27 (0.19; 0.31) μmoL/g Hb vs. 0.33 (0.20; 0.40) μmoL/g Hb, *p* = 0.04), GPO (4.08 (3.26; 4.60) mmoL/g Hb vs. 3.17 (2.68; 3.53) mmoL/g Hb, *p* = 0.02), and GR (21.22 (18.99; 25.40) mmoL/g Hb vs. 17.00 (15.78; 18.67) mmoL/g Hb, *p* = 0.04) levels were higher, compared with patients of group 1.

In the postoperative period in patients with POAF IL-6 (32.7 (23.8; 73.0) pg/mL vs. 20.1 (10.6; 50.8) pg/mL, *p* = 0.005), IL-8 (13.2 (10.5; 15.3) pg/mL vs. 5.9 (3.9; 8.8) pg/mL, *p* < 0.001), SOD (2267.6 (1542.4; 3299.8) units/g vs. 1098.3 (514.8; 2197.5) units/g, *p* < 0.001), RG (0.15 (0.13; 0.18) μmoL/g Hb vs. 0.27 (0.21; 0.32) μmoL/g Hb, *p* < 0.001), GPO (4.08 (3.26; 4.60) mmoL/g Hb vs. 3.17 (2.68; 3.53) mmoL/g Hb, *p* < 0.001) and GR (21.22 (18.99; 25.40) mmoL/g Hb vs. 17.00 (15.78; 18.67) mmoL/g Hb, *p* = 0.003) levels were higher, compared with patients without POAF.

No significant differences between the patients of the two groups were found according to other indicators. The data are shown in Table 3.

According to the results of the ROC analysis, we evaluated the sensitivity and specificity of different indicators included in the study (Table 4).

The highest sensitivity was observed for plasma SOD >1100.5 U/g (97.7%, *p* < 0.0001), the highest specificity—for GR after surgery ≤2.99 mmoL/g of hemoglobin (95%, *p* < 0.0001), the highest positive likelihood ratio was observed for the omega-3 index in the postoperative period (+LR = 6.8) (for patients with POAF, the probability of determining the omega-3 index in the postoperative period ≤1.59 is 6.8 times higher compared to the group without POAF), and the highest negative likelihood ratio was observed for the postoperative plasma SOD concentration (−LR = 0.04) (in patients without POAF, the probability of detecting plasma SOD after surgery >1100.5 U/g is 0.04 times higher compared to the group with POAF).

The results of univariate and multivariate regression analysis are shown in Table 5.

**Figure 2:**
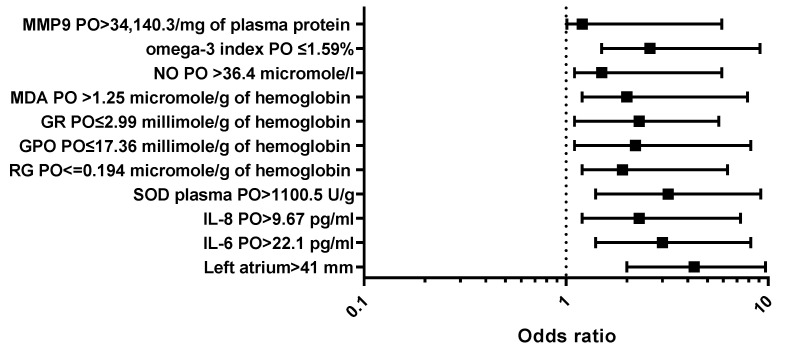
Factors associated with POAF (multivariate analysis). PO—postoperative.

In Figure 2 are shown the results of the multivariate analysis.

## 4. Discussion

AF usually develops during the first week after the intervention. As a rule, the arrhythmia is terminated within 24–48 h, and most of the patients are discharged with a sinus rhythm [7]. In our study, the new-onset POAF occurred on average 5.2 [2.0; 7.0] days after operation.

Our study demonstrated that patients with POAF were older, and according to the multivariate analysis, the patient’s age turned out to be an independent predictor of arrhythmia development. The same result was obtained by other authors [8]. Advanced age is a frequently identified risk factor for arrhythmia, which is explained by degenerative changes in the atria, which progress with age [9].

In our study, the increase in the left atrium diameter was associated with the risk of AF development [10]. The obtained data coincide with the results of Hung LT et al. (2021) [11] but differ from the opinion of Jakubova M. et al. (2012) [12]. According to the literature data, the involvement of the left atrium can be explained by an increase in fibrosis development with subsequent atrial dilatation. These changes are caused by indicators such as age-related aspects, mechanical damage, volume overload, intraoperative ischemia, electrolyte imbalance, hypertension and pericardial damage [13].

The mechanism of occurrence of POAF is determined as multifactorial and includes inflammatory response, oxidative stress, excessive production of catecholamines, dysfunction of the autonomic nervous system, accumulation of interstitial fluid as a result of changes in its volume and pressure and neurohormonal activation.

In our study, an increase in CRP, NT-proBNP and troponin levels were noted during CABG. Nevertheless, the above-mentioned levels did not differ significantly when comparing groups with POAF and without arrhythmia, which is also shown by other authors [14,15,16].

In the present study, during routine CABG, there was a significant increase in the concentration of IL-6 and IL-8 in the group of patients with arrhythmia, which corresponds to the systematic review and meta-analysis results by Weymann A. et al. (2013) [17] and differs from the study of Bjorgvinsdottir L. et al. (2013) [18]. As many potential causes of POAF are associated with postsurgical inflammation, increased levels of inflammatory biomarkers particularly postoperative interleukin-6 are considered in detail. The role of these inflammatory markers in the pathogenesis of POAF remains under vigorous investigation.

The present study shows a significant increase in concentration of MMP-9 in the group of patients with POAF, and this indicator, according to multivariate regression analysis, turned out to be an independent predictor of arrhythmia. Our results differ from the data of Bening C. et al. (2019), where the level of MMP-9 was reduced in the group of patients with AF that occurred after CABG [19].

Despite the importance of oxidative stress in the initiation of CVD, including AF, the role of key components of antioxidant protection needs further investigation. This concerns the glutathione system, which plays an important role in protecting cells from oxidative damage. In the present study, there was a significant decrease in RG and GPO, and an increase in GR during surgery in the group with POAF compared to the patients with sinus rhythm. These findings can be explained by severe impairments of the prooxidant–antioxidant balance and also by the systemic inflammatory response in patients who undergo CABG.

Oxidative stress is considered to be one of the mechanisms causing the occurrence of POAF. The development of oxidative stress in a number of pathologies is caused by an intensification of the processes of free radical oxidation and/or a decrease in the reserve of antioxidant protection, which causes oxidative modification of protein and lipid molecules, damage and disruption of the membrane structure, including apoptosis [20].

The present study revealed an increase in plasma SOD at the preoperative period, which indicates the activation of lipid peroxidation in patients with multivessel CAD. These findings are shown by Montaigne D. et al. (2013). The authors have shown that an increase in the concentration of reactive oxygen species before surgery predisposes to the occurrence of POAF [21].

In our study, plasma SOD level decreased after CABG. The decrease in plasma SOD concentration after myocardial revascularization indicates an intensification of oxidative stress during surgery and reflects increased consumption of the enzyme in patients with CAD. At the same time, SOD concentration in patients with POAF remains significantly higher compared to the group of patients with sinus rhythm, which may be due to its low activity when involved in the oxidation process. The presented results correspond to the data of Montaigne D. et al. (2013) [22].

In the present study, a significant increase in the level of MDA of erythrocytes was observed in patients with POAF, which, along with a decrease in the level of RG, GR and GPO, reflects the depletion of the antioxidant system of the cell against the background of increased activity of oxidative stress factors.

We found a decrease in omega-3 index, EPA and DHA in the erythrocyte membrane in patients after CABG, and this is consistent with the literature data [23].

Nowadays, it is necessary to estimate the factors of inflammation, oxidative stress, myocardial dysfunction, ischemia and fibrosis that are involved in the regulation of inflammation, oxidative component and fibrosis in patients with coronary artery disease. This may help in searching for highly sensitive markers associated with the occurrence of POAF, followed by the development of a prognostic scale using these indicators. The implementation of this scale into clinical practice will allow doctors to use these parameters to determine the risk of arrhythmia and to carry out differentiated pharmacotherapy depending on the identified risk category.

Thus, our study has limitations due to the small number of enrolled patients, which potentially affects the strength of our results. We did not analyze the influence of differences between pre- and postoperative levels of factors including inflammation, oxidative stress, fibrosis and omega-3 index in POAF development.

## 5. Conclusions

Our study showed that an increased left atrium diameter, high postoperative levels of inflammatory factors, oxidative stress, fibrosis indicators and omega-3 index were associated with postoperative atrial fibrillation in patients who underwent coronary artery bypass graft surgery.

## Figures and Tables

**Table 1:** Clinical characteristics of enrolled patients.

Indicators	Group 1 (n = 111)	Group 2 (n = 47)	*p*-Value
Male, n (%)	91 (82.0)	40 (84.4)	0.8
Age, years	62.0 (56.0; 66.0)	65.0 (61.0; 70.0) *	0.008
Smokers, n (%)	75 (67.6)	33 (70.2)	0.74
Body mass index > 30, n (%)	53 (47.7)	21 (44.7)	0.72
History of myocardial infarction, n (%)	71 (64.0)	30 (63.8)	0.99
History of CAD, months	15.5 (8.0; 72.0)	60.0 (13.5; 138.0) *	0.01
Hypertension, n (%)	110 (99.1)	47 (100.0)	0.66
Diabetes mellitus, n (%)	17 (15.3)	10 (21.3)	0.36
Transient ischemic attack/stroke, n (%)	9 (8.1)	4 (8.5)	0.58
Peripheral artery disease, n (%)	110 (99.0)	46 (98.0)	0.88
COPD, n (%)	86 (77.5)	39 (83.0)	0.57
Chronic kidney disease, n (%)	14 (12.6)	5 (10.6)	0.8
Medical treatment before surgery:			
β blockers, n (%)	89 (80.2)	38 (81.3)	0.9
angiotensin-converting enzyme inhibitors/angiotensin receptor blockers, n (%)	78 (70.3)	34 (72.3)	0.79
calcium channel blockers, n (%)	33 (29.7)	15 (31.9)	0.78
nitrates, n (%)	49 (44.1)	25 (53.2)	0.3
diuretics, n (%)	12 (10.8)	6 (12.8)	0.86
atorvastatin, n (%)	63 (56.8)	19 (40.4)	0.06
aspirin, n (%)	92 (83.0)	34 (72.3)	0.13
clopidogrel, n (%)	47 (42.3)	16 (34.0)	0.33

NYHA—New York Heart Association. *—*p* < 0.05

**Table 2:** Instrumental and surgical indicators of studied patients.

Indicators	Group 1 (n = 111)	Group 2 (n = 47)	*p*-Value
Left atrial diameter, mm	38.0 (36.0; 40.0)	44.0 (40.5; 46.0) *	<0.001
Left atrial volume, mL	44.1 (41.9; 45.4)	55.9 (48.1; 63.1) *	<0.001
Left ventricular end-systolic dimension, (mm)	34.0 (30.0; 40.0)	36.5 (32.0; 41.0)	0.19
Left ventricular end-diastolic dimension, (mm)	52.0 (48.0; 56.0)	53.0 (48.5; 59.5)	0.19
Left ventricular end-systolic volume, (mL)	51.0 (39.0; 65.0)	53.0 (44.0; 62.0)	0.5
Left ventricular end-diastolic volume, (mL)	123.0 (97.0; 137.0)	132.0 (113.0; 151.0)	0.19
Left ventricular ejection fraction, %	59.5 (50.0; 65.0)	58.0 (47.5; 62.0)	0.26
Glomerular filtration rate, mL/min/1.73 m^2^ (CKD-EPI)	74.0 (65.0; 86.0)	78.5 (66.0; 87.0)	0.44
EuroScore risk	1.1 (0.88; 2.3)	1.5 (1.03; 2.3)	0.098
Left coronary artery stenosis ≥ 50%, n (%)	17 (15.3)	13 (27.7)	0.07
Number of grafts			
1, n (%)	10 (9.1)	1 (2.1)	0.1
2, n (%)	36 (32.4)	15 (31.9)	0.95
3, n (%)	54 (48.6)	27 (57.4)	0.31
4, n (%)	11 (9.9)	4 (8.5)	0.52
Off-pump, n (%)	9 (15.3)	2 (9.1)	0.12
Time of extracorporeal circulation, min	58.0 (46.0; 69.0)	62.0 (50.0; 70.0)	0.3
Aortic cross-clamping time, min	33.0 (25.0; 42.0)	37.0 (30.0; 40.0)	0.6
Time of ischemia, min	13.0 (7.0; 18.0)	13.0 (9.0; 19.0)	0.4
Time of lung mechanical ventilation, min	525.0 (405.0; 655.0)	580.0 (465.0; 728.0)	0.068

*—*p* < 0.05.

**Table 3:** Clinical and instrumental indicators of studied patients.

Indicators	Group 1 (n = 111)		Group 2 (n = 47)	
	Before Operation	After Operation	Before Operation	After Operation
IL-6, pg/mL	3.6 (2.6; 15.6)	20.1 (10.6; 50.8) #	5.5 (3.5; 46.0) *	32.7 (23.8; 73.0) **, ##
IL-8, pg/mL	1.7 (1.2; 2.7)	5.9 (3.9; 8.8) #	1.7 (1.1; 3.5)	13.2 (10.5; 15.3) **, ##
NT-proBNP, pg/mL	113.6 (24.4; 271.1)	424.0 (202.3; 532.5) #	101.9 (40.05; 206.0)	473.0 (253.0; 868.0) ##
Troponin I, mkg/L	-	1.6 (1.1; 3.0)	-	2.0 (0.94; 2.5)
CRP, g/L	0.8 (0.3; 1.6)	4.7 (4.2; 5.5) #	1.2 (0.2; 2.7)	4.5 (4.2; 6.1) ##
SOD of plazma, U/g	1775.8 (924.8; 4718.8)	1098.3 (514.8; 2197.5) #	2751.8 (1563.0; 3949.8) *	2267.6 (1542.4; 3299.8) **,##
RG, μmoL/g Hb	0.33 (0.20; 0.40)	0.27 (0.21; 0.32) #	0.27 (0.19; 0.31) *	0.15 (0.13; 0.18) **,##
GPO, mmoL/g Hb	4.08 (3.26; 4.60)	4.18 (3.76; 4.73)	3.17 (2.68; 3.53) *	2.99 (2.93; 3.99) **
GR, mmoL/g Hb	21.22 (18.99; 25.40)	21.06 (19.34; 24.53)	17.00 (15.78; 18.67) *	16.35 (14.96; 16.72) **,##
MDA, μmoL/g Hb	0.32 (0.26; 0.38)	0.37 (0.24; 0.48) #	0.33 (0.22; 0.44)	1.98 (1.32; 2.38) **,##
NO, μmoL/g Hb	40.6 (30.4; 45.2)	36.0 (30.8; 47.9)	31.8 (25.8; 52.2)	50.8 (39.5; 70.1) **,##
MMP-9/mg of plasma protein	14,171.6 (12,165.9; 20,412.9)	17,625.2 (12,217.3; 22,325.3)	23,914.6 (17,524.9; 31,799.7) *	45,015.7 (38,495.8; 66,652.7) **,##
Eicosapentaenoic acid (C 20:5), %	0.72 (0.51; 1.05)	0.72 (0.48; 0.94)	0.27 (0.00; 0.89)	0.00 (0.00; 0.21) **,#
Docosahexaenoic acid (C 22:6), %	5.93 (3.38; 6.57)	5.01 (3.45; 6.57)	2.52 (0.63; 4.52) *	0.30 (0.19; 0.83) **,#
Omega-3 index, %	5.38 (4.10; 7.62)	5.01 (3.91; 7.2)	2.01 (0.75; 5.50) *	0.36 (0.15; 1.06) **,#
Missing values	Group 1	(n = 44)	Group 2	(n = 18)

Note. *—*p* I-II before surgery <0.05; **—*p* I-II after surgery <0.05; #—*p* I before and after surgery <0.05; ##—*p* II before and after surgery <0.05.

**Table 4:** Sensitivity (Se) and specificity (Sp) of the indicators included in the study.

Indicators	AUC	Se, %	Sp, %	+LR	−LR	*p*-Value
Age >62 years	0.75	77	62	2.03	0.37	<0.001
Stable angina III-IV	0.6	73	48	1.4	0.57	0.02
NYHA III	0.63	34	94	5.68	0.7	0.001
Period of CAD >20 months	0.62	68	54	1.48	0.59	0.03
Left atrium >41 mm	0.86	73	89	6.61	0.33	<0.001
IL-6 after CABG >22.7 pg/mL	0.68	81.8	60	2.1	0.03	<0.001
IL-8 after CABG >9.67 pg/ml	0.72	81.8	66	2.41	0.28	<0.001
SOD in plazma after CABG >1100.5 U/g	0.77	97.7	52	2.04	0.04	<0.001
RG after CABG ≤0.194 μmoL/g Hb	0.88	93.2	75	3.73	0.091	<0.001
GPO before CABG ≤18.7 mmoL/g Hb	0.915	84	86	6.01	0.18	<0.001
GPO after CABG ≤17.36 mmoL/g Hb	0.92	91	88	6.4	0.12	<0.001
GR before CABG ≤3.6 mmoL/g Hb	0.73	93.2	59	2.3	0.12	<0.001
GR after CABG ≤2.99 mmoL/g Hb	0.81	56.8	95	5.8	0.43	<0.001
MDA after CABG >1.25 μmoL/g Hb	0.9	81.8	85	5.5	0.21	<0.001
MMP-9 after CABG >34,140.3/mg of plasma protein	0.99	93.2	94	4.5	0.1	<0.001
NO after CABG >36.4 μmoL/g Hb	0.6	84.1	50	1.68	0.32	0.04
Omega-3 index before CABG ≤2.6%	0.81	65.9	90	6.6	0.38	<0.001
Omega-3 index after CABG ≤1.59%	0.84	68.6	92	6.8	0.27	<0.001

AUC—area under the ROC curve; +LR—positive likelihood ratio; −LR—negative likelihood ratio.

**Table 5:** Indicators of logistic regression analysis.

Indicators	Univariate Regression Analysis			Multivariate Regression Analysis		
	OR	95% CI	*p*-Value	OR	95% CI	*p*-Value
age >62 years	2.1	1.04–4.6	0.03			
stable angina III-IV class	1.6	0.98–2.6	0.05			
NYHA III	3.6	2.3–5.7	0.001			
history of CAD >20 months	2.9	1.4–6.2	0.004			
left atrium diameter >41 mm	5.3	2.2–8.5	<0.001	4.3	2.0–9.7	<0.001
IL-6 postoperative levels >22.07 pg/mL	3.4	2.3–7.3	0.001	3.0	1.4–8.2	0.006
IL-8 postoperative levels >9.67 pg/mL	2.8	1.8–4.7	<0.001	2.3	1.2–7.3	0.006
SOD postoperative levels in plasma >1100.5 U/g	4.7	1.4–7.7	<0.001	3.2	1.4–9.2	0.03
RG postoperative levels ≤0.194 micromole/g of hemoglobin	2.4	1.3–4.9	0.001	1.9	1.2–6.3	<0.001
GPO preoperative levels ≤18.7 millimole/g of hemoglobin	1.6	1.1–5.8	0.001			
GPO postoperative levels ≤17.36 millimole/g of hemoglobin	2.7	1.2–6.2	<0.001	2.2	1.1–8.2	<0.001
GR preoperative levels ≤3.6 millimole/g of hemoglobin	1.7	1.1–4.9	0.001			
GR postoperative levels ≤2.99 millimole/g of hemoglobin	2.9	1.2–3.9	<0.001	2.3	1.1–5.7	<0.001
MDA postoperative levels >1.25 micromole/g of hemoglobin	2.5	1.5–5.7	<0.001	2.0	1.2–7.9	<0.001
MMP-9 postoperative levels >34,140.3/mg of plasma protein	1.7	1.05–4.1	0.01	1.2	1.01–5.1	0.001
NO postoperative levels in plasma >36.4 micromole/l	2.1	1.4–3.8	<0.001	1.5	1.1–5.9	<0.001
omega-3 index preoperative levels ≤2.6%	1.8	1.2–5.1	<0.001			
omega-3 index postoperative levels ≤1.59%	3.1	1.9–6.2	<0.001	2.6	1.5–9.1	<0.001

## Data Availability

No new data were created or analyzed in this study. Data sharing is not applicable to this article.
